# Comparison between the trapezius and adductor pollicis muscles as an acceleromyography monitoring site for moderate neuromuscular blockade during lumbar surgery

**DOI:** 10.1038/s41598-021-94062-2

**Published:** 2021-07-15

**Authors:** Seok Kyeong Oh, Sangwoo Park, Byung Gun Lim, Young Sung Kim, Heezoo Kim, Myoung Hoon Kong

**Affiliations:** Department of Anesthesiology and Pain Medicine, Korea University Guro Hospital, Korea University College of Medicine, Seoul, Republic of Korea

**Keywords:** Musculoskeletal system, Outcomes research, Medical research, Randomized controlled trials

## Abstract

Acceleromyography at the adductor pollicis located in a distal part of the body may not reflect the degree of neuromuscular blockade (NMB) at the proximally located muscles manipulated during lumbar surgery. We investigated the usefulness and characteristics of acceleromyographic monitoring at the trapezius for providing moderate NMB during lumbar surgery. Fifty patients were randomized to maintain a train-of-four count 1–3 using acceleromyography at the adductor pollicis (group A; n = 25) or the trapezius (group T; n = 25). Total rocuronium dose administered intraoperatively [mean ± SD, 106.4 ± 31.3 vs. 74.1 ± 17.6 mg; *P* < 0.001] and surgical satisfaction (median [IQR], 7 [5–8] vs. 5 [4–5]; *P* < 0.001) were significantly higher in group T than group A. Lumbar retractor pressure (88.9 ± 12.0 vs. 98.0 ± 7.8 mmHg; *P* = 0.003) and lumbar muscle tone in group T were significantly lower than group A. Time to maximum block with an intubating dose was significantly shorter in group T than group A (44 [37–54] vs. 60 [55–65] sec; *P* < 0.001). Other outcomes were comparable. Acceleromyography at the trapezius muscle during lumbar surgery required a higher rocuronium dose for moderate NMB than the adductor pollicis muscle, thereby the consequent deeper NMB provided better surgical conditions. Trapezius as proximal muscle may better reflect surgical conditions of spine muscle.

## Introduction

As the relationship between the degree of neuromuscular blockade (NMB) and the surgical environment has been reported^1–5^, it is important to accurately measure the degree of NMB intraoperatively.

In clinical practice, for most patients requiring general anesthesia, NMB monitoring is performed using the adductor pollicis muscle. However, acceleromyography at the adductor pollicis muscle may not reflect the degree of NMB at the proximally located muscles manipulated during lumbar spine surgery as this muscle belongs to the distal part of the body^6^. NMB recovery at the proximal muscles such as laryngeal and diaphragmatic muscles is more rapid than that at the adductor pollicis muscle^7–9^; thus, acceleromyography at the latter muscle may be unreliable to assess the degree of NMB at the proximal muscles and estimate the optimal muscle relaxant dose needed for proximal muscle NMB. Recent studies have shown that neuromuscular monitoring at the trapezius can be an acceptable alternative to that at the adductor pollicis muscle^10,11^. Therefore, as the trapezius is a proximal muscle, acceleromyography at this muscle may be better than that at the adductor pollicis muscle for NMB monitoring during lumbar surgery.

Herein, we investigated the usefulness and characteristics of NMB monitoring using acceleromyography at the trapezius by assessing the following: total rocuronium dose administered intraoperatively, time to maximum block with an intubating dose, intubating conditions, lumbar retractor pressure, degree of lumbar muscle tone, overall surgical satisfaction score, and the postoperative pain score between the patient groups with acceleromyography at the two different measurement sites for providing moderate NMB (train-of-four [TOF] count 1–3) during lumbar surgery. We hypothesised that neuromuscular monitoring at the trapezius would require a higher rocuronium dose than that required for neuromuscular monitoring at the adductor pollicis muscle to maintain moderate NMB intraoperatively, which would provide better surgical conditions.

## Methods

### Patients and study design

**Figure 1:**
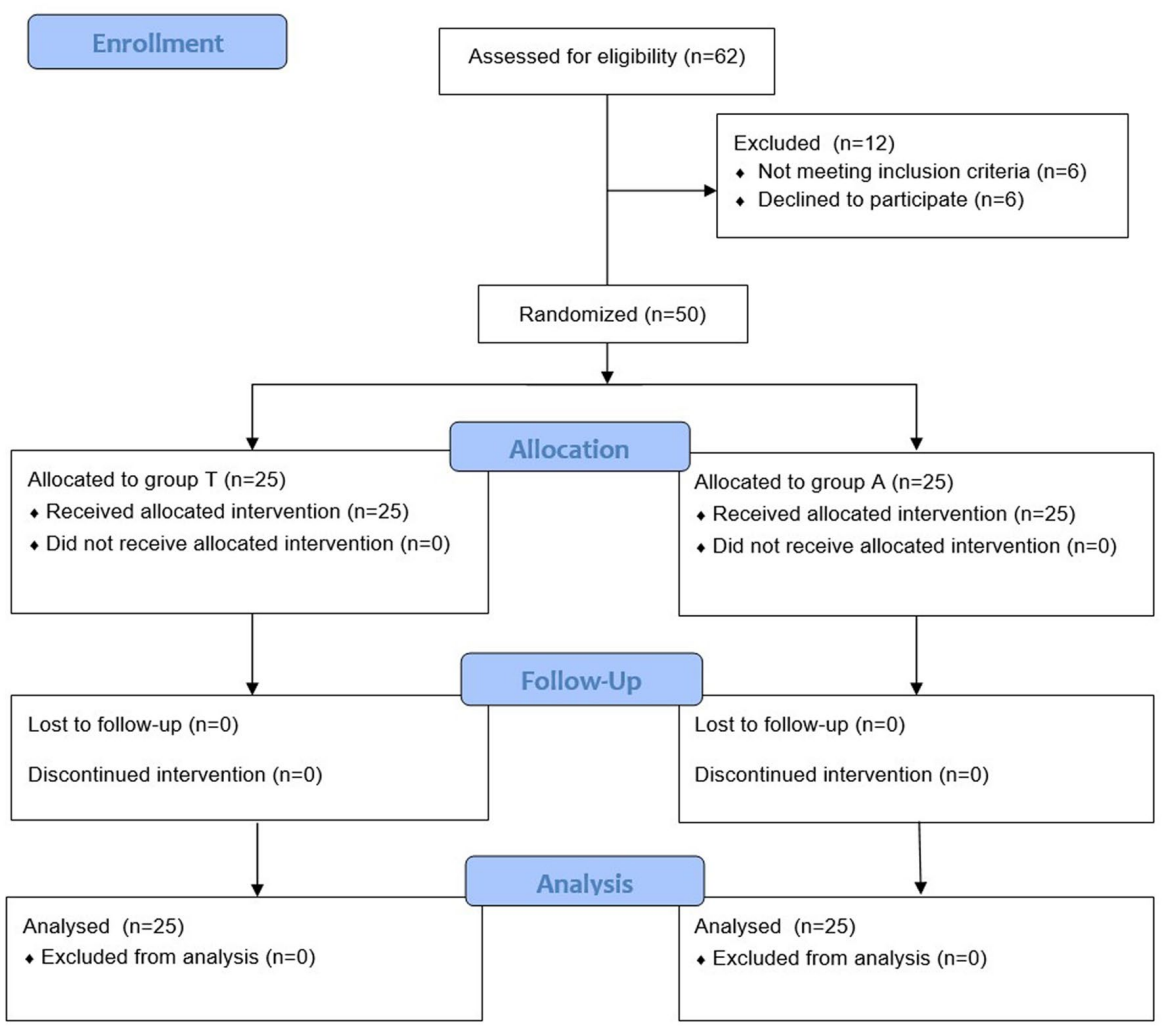
Flow-chart describing patient recruitment, randomization, and withdrawal. Group T: Trapezius muscle group maintaining TOF (train-of-four) count 1–3 based on acceleromyography at the trapezius muscle. Group A: Adductor pollicis muscle group maintaining TOF count 1–3 based on acceleromyography at the adductor pollicis muscle.

This single-centre prospective double-blinded randomised controlled trial followed the Consolidated Standards of Reporting Trials (CONSORT) 2010 guidelines (Fig. 1), and was conducted at Korea University Guro Hospital, Seoul, South Korea, from October 2018 to June 2019. Patients aged 19–75 years, with ASA physical status I–II, undergoing elective lumbar spine surgery were recruited. Patients were excluded if they had a known hypersensitivity to the drugs used in this study, known neuromuscular disease, significant hepatic or renal dysfunction, cerebrovascular disease, or a body mass index > 30.0 kg m^−2^. Patients who required intraoperative neurophysiological monitoring, including motor evoked potential, were also excluded.

Participants were randomly assigned to either the trapezius muscle group (group T) or the adductor pollicis muscle group (group A) by a web-based computer-generated list; they were unaware of their assignment. The randomized numbers were kept in opaque, sealed envelopes and were opened in the operating room by an independent anesthesiologist. The investigators who assessed the study endpoints associated with surgical or intubating conditions were blinded to the group allocation. In order to blind investigator including surgeons, we covered each the trapezius and adductor pollicis muscle using antiseptic cloth and applied acceleromyography on both the trapezius muscle and the adductor pollicis muscle. The mayo stand was used to support the antiseptic cloth so as not to interfere with the free movement of the trapezius and adductor pollicis muscle. Only the independent anesthesiologist who performed anesthesia maintenance and NMB management was unblinded.

### Anesthetic regimen and NMB management

All patients were monitored with electrocardiography, non-invasive blood pressure, pulse oximetry, and capnography. Hypnosis depth was monitored using the bispectral index (BIS; Aspect Medical Systems, Norwood, MA, USA). NMB, following anesthesia induction, was assessed with the TOF-Watch SX (Organon Ireland Ltd, Schering-Plough Corporation, Dublin, Ireland), applied at both the left trapezius muscle and the right adductor pollicis muscle in all patients. NMB monitoring and management were performed according to the Good Clinical Research Practice guidelines^12^.

NMB monitoring was performed at both the adductor pollicis and trapezius sites in all patients to partially blind the study, regardless of the group allocation. But only one set of results, which corresponded to the group allocation, was used in the data analysis for each patient.

For the adductor pollicis muscle monitoring, electrodes were attached to the ulnar nerve passage, and the acceleration transducer with an elastic preload (Hand Adapter, Organon) was fixed to the distal portion of the thumb. The wrist was immobilised with a detachable wrist brace (Neoban wrist support, Seoul Brace, Seoul, South Korea)^13^, which permitted free thumb movement. The wrist brace was applied for improving accuracy of TOF-watch SX monitoring in the prone position by moving the thumb freely but fixing the other parts of the hand.

**Figure 2:**
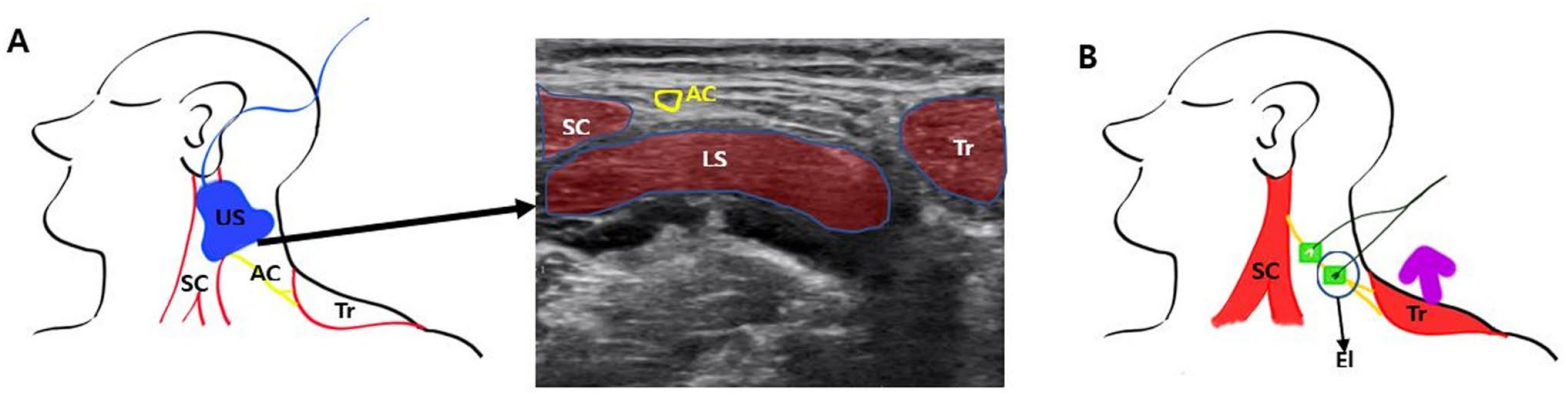
Illustrations and ultrasound image of methods for detection of the accessory nerve and the placement of the electrodes and transducer of the accelerometer in the trapezius muscle group (group T). A: The accessory nerve was confirmed by ultrasonography; the picture indicates the accessory nerve and surrounding muscles. B: The electrodes were placed over the accessory nerve. The purple arrow indicates a cranial direction of shoulder movement by electrical stimulation (shrug). The electrodes for acceleromyography were placed on the accessory nerve after the pathway was confirmed by ultrasonography. The transducer of the accelerometer was fixed at the left trapezius muscle at approximately 10 cm from the electrodes. SC: Sternocleidomastoid muscle, Tr: Trapezius muscle, AC: Yellow line, accessory nerve, US: Ultrasonography, El: Electrodes LS: Levator scapulae muscle.

For the trapezius muscle monitoring, the accessory nerve passage was confirmed using ultrasonography (SONIMAGE HS1, Konica Minolta Inc, Japan), and electrodes were placed over the left accessory nerve, with a 2 cm distance between the electrodes (Fig. 2). The acceleration transducer was fixed using a fixing product (Multifix, Unimedics, Seoul, South Korea) over the left trapezius approximately 10 cm distal from the electrodes. Appropriate accessory nerve stimulation was verified with the left shoulder movement (‘‘shrug’’) in the cranial direction^10,11^.

General anesthesia was induced and maintained with total intravenous anesthesia using propofol-remifentanil. The effect-site concentration of propofol was increased stepwise from 3.0 µg ml^−1^ until a BIS of < 60. When BIS reached < 60 and the eyelash reflex was lost, the TOF-Watch SX was calibrated using an automatic calibrating procedure, and TOF stimuli were commenced at both sites. Then, stabilization, followed by re-calibration, were performed; the TOF ratio measured after the re-calibration was the baseline value^12,14^. During these procedures, the BIS values were maintained at 40–60, and an independent anesthesiologist maintained face mask ventilation with an oropharyngeal airway, and the patient’s head was kept in a neutral position to stabilize the sternocleidomastoid and trapezius muscles. Rocuronium 0.5 mg kg^−1^ was subsequently injected, and endotracheal intubation was performed after confirming maximum block. TOF measurement was repeated every 15 s. The rocuronium infusion solution contained rocuronium (1 mg ml^−1^) diluted in normal saline. The rocuronium infusion was started at 0.4 mg kg^−1^ h^−1^ immediately after TOF count 1, and the rate was adjusted to maintain a TOF count of 1–3 of acceleromyography at the trapezius in group T, and that of 1–3 of acceleromyography at the adductor pollicis muscle in group A. The effect-site concentration of propofol and remifentanil was titrated to maintain a BIS value of 40–60, and mean arterial pressure within ± 30% from the baseline, respectively.

### Assessment of intubating conditions

Endotracheal intubation was performed after confirming maximum block from rocuronium 0.5 mg kg^−1^ at the adductor pollicis muscle in group A and at the trapezius muscle in group T. A single experienced anesthesiologist performed all intubations. The intubating conditions were evaluated by the following three variables: convenience of laryngoscopy, position and movement of the vocal cords, and diaphragmatic response, including cough or limb movement during intubation or immediately after intubation^12^. The overall intubating conditions were rated excellent if all variables were excellent, good if all variables were good or excellent, and poor if any variable was poor.

### Evaluation of surgical conditions

An experienced spine surgeon, blinded to the group allocation, performed the following procedures to minimise possible heterogeneity. A large screen was established for masking groups from the surgeons just after placing the patient prone^13^. Before suturing the fascia at the end of the surgical procedure, a flat planar pneumatic pressure transducer (Catheter ICP monitor probe, Spiegelberg GmbH, Hamburg, Germany) was placed between the surgical lumbar retractor blade (Karlin™ Crank Frame Spinal Retractor Set, Codman, Germany) and the multifidus muscle. Then, the lumbar retractor blades were retracted until a full view of the operative lumbar level was obtained^13^. An independent anesthesiologist then measured the pressure on a pneumatic pressure transducer as the lumbar retractor pressure. The detailed technique of measuring the lumbar muscle retractor pressure can be found in our previous study^13^.

The surgeon evaluated the lumbar muscle tone during surgery in three categories (good, suitable for the suture; moderate, not affecting the suture; and hard, making the suture difficult) just before suturing the fascia. The surgeon assessed the overall surgical satisfaction score using a 10-point scale (1–10; 1 = extremely poor; 10 = excellent) postoperatively.

### Postoperative recovery, pain management, and analgesic regimen

Fentanyl 50 µg and ramosetron 0.3 mg were injected at the end of the surgery. The patients were returned to the supine and propofol-remifentanil infusion was discontinued. In all patients, NMB reversal was performed with sugammadex based on the TOF of acceleromyography at the adductor pollicis muscle, regardless of the group assignment. For TOF count 0 and post-tetanic count ≥ 1, sugammadex 4 mg kg^−1^ was used; for TOF count 1–3, sugammadex 2 mg kg^−1^ was used. Extubation was performed with tidal volume > 5–10 ml kg^−1^, and TOF ratio of > 0.9 at the adductor pollicis muscle. Supplementary Figure S1 shows an example of the neuromuscular monitoring at each measurement site.

In the PACU, the pain score was evaluated using a NRS (1–10) every 10 min, and fentanyl 10 µg was administered for NRS > 3. Intravenous PCA; fentanyl, 800 µg; nefopam, 200 mg; total volume, 60 ml with normal saline; basal infusion rate, 0.5 ml h^−1^ with a bolus dose of 0.5 ml and a 15-min lockout period) was started immediately after patients’ arrival at the PACU^15,16^. The sedation score was assessed by the Modified Observer’s Assessment of Awareness/Sedation Score (MOAA/SS)^17^. The patients were transferred to the ward when the NRS < 4 and the MOAA/SS was 5, and they were observed until 48 h postoperatively.

### Study outcomes

The primary outcome of the study was the total rocuronium dose administered intraoperatively, and the other outcomes were the secondary outcomes.

#### Anesthesia induction and intraoperative outcomes

The measured outcomes included: (1) Total rocuronium dose administered intraoperatively (primary outcome); (2) time to maximum block with an intubating dose; (3) intubating conditions; (4) lumbar retractor pressure measured immediately before fascia suture; (5) lumbar muscle tone (good/moderate/hard); (6) overall surgical satisfaction score assessed by the surgeon; (7) sugammadex dose for NMB reversal; (8) average infusion rate of propofol and (9) remifentanil; (10) time to extubation from the end of the infusion of anesthetics.

#### Postoperative outcomes

The postoperative outcomes included: (1) length of PACU stay; (2) rescue fentanyl consumption in the PACU; (3) total PCA consumption until 48 h postoperatively in the ward; (4) NRS score for pain (1–10) on arrival in the PACU, and 30 and 60 min after arriving in the PACU, and at 6, 24, and 48 h postoperatively; and (5) the occurrence of adverse events (e.g., respiratory depression, over-sedation, hypersensitivity, tachycardia, bradycardia, hyper- or hypotension, headache, dizziness, urination difficulty, and nausea and vomiting) in the PACU and in the ward until 48 h postoperatively.

### Statistical analysis

The sample size calculation was based on a pilot study with five cases in each group with G*Power 3 software^18^. In the pilot study, mean ± SD of total rocuronium dose administered intraoperatively was 78.0 ± 22.9 mg in group A and 103.8 ± 35.3 mg in group T. The effect size was 0.867. Assuming an allocation ratio of 1:1, 22 patients were selected for each group, calculated using a two-sided Student’s t-test with a significance level of 0.05 and a power of 0.8. We estimated a 10% dropout, resulting in enrolment of 25 patients in each group.

Statistical analyses were performed using the SPSS software (version 20.0; IBM, Armonk, NY, USA). Continuous variables were compared using the Student’s t-test or the Mann–Whitney U-test. Ordinal parameters were compared using the Mann–Whitney U-test, and categorical variables were compared by a chi-square test. Changes in the NRS sore for pain in the PACU and in the ward were compared using repeated measures analysis of variance.

Data are expressed as mean ± SD, for normally distributed variables, median (range) or median [IQR] for non-normally distributed variables or ordinal variables, and absolute number for categorical variables. *P* values were two-tailed, and *P* < 0.05 was considered statistically significant. For the multiple comparison of 14 secondary outcomes, adjusted alpha (α / k [number of hypothesis test]) corrected values using Bonferroni correction were applied^19^, thereby *P* < 0.0036 was considered statistically significant.

### Ethics approval

The Institutional Review Board of Korea University Guro Hospital, Seoul, South Korea approved this single-centre prospective double-blinded randomised controlled trial (IRB No.: 2018GR0255). This study registered at UMIN clinical trials registry (Identifier: UMIN000038309, date of registration: 01/11/2019) and was conducted in accordance with the guidelines of Good Clinical Practice. All patients provided written informed consent.

### Disclosure

This manuscript in part has been presented as an e-poster at the Virtual Euroanaesthesia 2020 (28–30 Nov. 2020) and has been published in the e-Supplement to the European Journal of Anaesthesiology (Volume 37, e-Supplement 58, June 2020).

## Results

Sixty-two patients were enrolled, and 12 patients were excluded as they did not meet the inclusion criteria or declined participation. Finally, 50 patients were assigned to two groups and evaluated (Fig. 1). The patients’ characteristics, surgical and anesthesia times, and the number of operated spinal levels were comparable between the two groups (Table 1).

**Table 1:** Demographic and clinical characteristics of patients. *ASA* American Society of Anesthesiologists; *SD* standard deviation; *IQR* interquartile range.

	Trapzius (n = 25)	Adductor pollicis (n = 25)	*P* value
Age, median (range) (y)	64 (24–73)	61 (40–72)	0.547
Sex (male), n (%)	8 (32%)	10 (40%)	0.769
Height, mean ± SD (m)	1.61 ± 0.08	1.61 ± 0.09	0.934
Weight, median [IQR] (kg)	63 [60–67]	67 [59–70]	0.361
**ASA class, n (%)**			0.561
I	8 (32%)	11 (44%)	
II	17 (68%)	14 (56%)	
**Operation level, n (%)**			0.762
1	18 (72%)	16 (64%)	
2	7 (28%)	9 (36%)	
Surgical time, median [IQR] (min)	120 [74–150]	155 [100–190]	0.127
Anesthesia time, mean ± SD (min)	207.2 ± 57.6	221.9 ± 66.9	0.409

The median [IQR] time to maximum block with an intubating dose was significantly shorter in the trapezius muscle group (group T) than in the adductor pollicis muscle group (group A) (44 [37–54]) vs. 60 [55–65] sec; *P* < 0.001). However, the intubating conditions were not significantly different between the two groups (Table 2).

**Table 2:** Intraoperative outcomes, including intubating conditions, time to maximum block for an intubating dose, and rocuronium dose for induction. IQR indicates interquartile range.

	Trapezius (n = 25)	Adductor pollicis (n = 25)	*P* value
**Variables for intubating conditions, n**			
Convenience of laryngoscopy			
Easy/fair/difficult	24/1/0	23/2/0	0.552
Position and movement of the vocal cords			
Abducted/intermediate/closed	22/0/3	22/0/3	1.000
Diaphragmatic or limb movements during intubation or immediately after intubation			
None/slight/sustained	11/14/0	14/11/0	0.396
**Overall intubating conditions**			
Excellent/good/poor	10/12/3	14/8/3	0.480
Time to maximum block, median [IQR] (sec)	44 [37–54]	60 [55–65]	< 0.001
Rocuronium for induction, median [IQR] (mg)	31 [30–34]	34 [30–35]	0.441

The total rocuronium dose administered intraoperatively was significantly higher in the group T than in the group A (mean ± SD; 106.4 ± 31.3 vs. 74.1 ± 17.6 mg; *P* < 0.001). Sugammadex dose for NMB reversal was significantly higher in the group T than in the group A (240 [230–260] vs. 130 [120–140]) mg; *P* < 0.001). Sugammadex 4 mg kg^−1^ was used for NMB reversal in 22 patients in the group T and 0 patient in the group A (*P* < 0.001). The lumbar retractor pressure was significantly lower in the group T than in the group A (88.9 ± 12.0 vs. 98.0 ± 7.8 mmHg; *P* = 0.003). The lumbar muscle tone and overall surgical satisfaction score evaluated by the surgeon (7 [5–8]) vs. 5 [4, 5]; *P* < 0.001) in the group T were superior to those in the group A. The average propofol infusion rate and the average remifentanil infusion rate showed no statistical difference. Time to extubation from the end of the infusion of anesthetics did not differ significantly between the two groups (Table 3).

**Table 3:** Intraoperative outcomes, including variables measured for surgical condition assessment, cumulative dose of rocuronium, average infusion rate of anesthetics, and sugammadex dose for reversal of NMB. NMB indicates neuromuscular blockade; *SD* standard deviation; *IQR* interquartile range.

	Trapezius (n = 25)	Adductor pollicis (n = 25)	*P* value
**Variables for surgical condition**			
Lumbar retractor pressure, mean ± SD (mmHg)	88.9 ± 12.0	98.0 ± 7.8	0.003
Lumbar muscle tone, n (good/moderate/hard)	13/10/2	1/18/6	< 0.001
Overall surgical satisfaction score, median [IQR] (1–10)	7 [5–8]	5 [4–5]	< 0.001
**Administered dose of drugs**			
Total rocuronium, mean ± SD (mg)	106.4 ± 31.3	74.1 ± 17.6	< 0.001
Infused rocuronium, mean ± SD (mg)	73.6 ± 29.6	41.0 ± 18.3	< 0.001
Sugammadex dose for reversal of NMB, median [IQR] (mg)	240 [230–260]	130 [120–140]	< 0.001
Remifentanil average rate, median [IQR] (μg kg^−1^ min^−1^)	0.059 [0.050–0.074]	0.054 [0.041–0.068]	0.367
Propofol average rate, mean ± SD (mg kg^−1^ min^−1^)	0.107 ± 0.045	0.133 ± 0.026	0.018
Time to extubation from the end of the infusion of anesthetics, median [IQR] (min)	15 [13–20]	16 [12–20]	0.900

In the post-anesthesia care unit (PACU), the length of stay (70 [60–85] vs. 70 [60–85] min; *P* = 0.929) and rescue fentanyl consumption (40 [10–50] vs. 40 [30–50] μg; *P* = 0.301) were not different between the group T and group A. The longitudinal changes in the numerical rating scale (NRS) score for pain (1–10) on arrival in the PACU, and 30 and 60 min after arriving in the PACU (5.6 ± 1.8, 4.3 ± 1.7, 2.7 ± 0.9 vs. 6.2 ± 1.7, 4.7 ± 1.6, 2.8 ± 1.1; *P* = 0.294) were comparable between the group T and group A. As an adverse event, hypotension occurred in one case in the group T, but no events in the group A.

In the ward, the longitudinal changes in the NRS score for pain at 6, 24, and 48 h postoperatively (4.1 ± 1.0, 2.7 ± 0.9, 1.7 ± 0.6 vs. 3.7 ± 1.0, 2.3 ± 0.6, 1.5 ± 0.6; *P* = 0.590) did not differ significantly between the group T and group A. The cumulative patient-controlled analgesia (PCA) consumption (46.3 ± 9.7 vs. 45.0 ± 11.8 ml; *P* = 0.659) and the incidence of nausea and vomiting as adverse events in the ward (2 vs. 1; *P* = 0.552) were not significantly different between the group T and group A.

## Discussion

In this study, neuromuscular monitoring at the trapezius offered several benefits compared to that at the adductor pollicis muscle during spine surgery. A main benefit is that it enables NMB monitoring of the proximal muscles, such as lumbar spine muscles, to be measured as closed to the actual value during spine surgery, given the superior surgical condition in the trapezius group. Moderate NMB maintenance (TOF count 1–3) based on the trapezius required infusion of relatively high rocuronium dose than that required for NMB maintenance based on the adductor pollicis muscle.

The proximally located muscles including the diaphragm and larynx are more resistant to the effect of neuromuscular blocking agents, and recover faster than peripheral muscles^6,9,20,21^. The proximally located muscles, including the diaphragm and larynx, have higher regional blood flows supplied by larger arteries branched from the aorta^20,21^. In addition, the peripheral (distal) muscles have a lower density of acetylcholine receptors than that in the proximal muscles^22^. Hence, both the onset and offset of proximal muscle NMB were faster than those of the distal muscles. Thus, the proximal muscles require a higher rocuronium dose to maintain the same degree of NMB as that to the distal muscles; this result is consistent with our study. In our study, higher sugammadex dose following higher rocuronium dose was needed to reverse the NMB in the group T than group A. Only the sugammadex dose (2 mg kg^−1^) needed for moderate NMB reversal might have resulted in incomplete NMB recovery in group T^8^. Considering this risk, we ensured patient safety in this study by administering the sugammadex dose for reversal of the actual NMB based on the adductor pollicis muscle in both groups. Consequently, the sugammadex dose needed for NMB reversal at the end of the surgery was 4 mg kg^−1^ in 22 patients (88%) in group T, but no patient (0%) in group A. In most patients in group T, maintaining moderate NMB based on the acceleromyography at the trapezius provided deep NMB based on that of the adductor pollicis muscle. Considering the better surgical outcomes with deeper NMB in group T than in group A, it is inferred that deep NMB may provide better surgical condition than moderate NMB although no study has investigated the effects of deep vs. moderate NMB during lumbar surgery. A recent study^23^ investigated the effects of deep vs. moderate NMB on surgical condition during lumbar surgery, and reported that the surgeon’s satisfaction score with the intraoperative surgical conditions was significantly higher in deep NMB than in moderate NMB, which is in line with our result.

In previous studies on laparoscopic surgery, a deep NMB improved intraoperative surgical conditions^1–5^. These findings may extend to lumbar surgery because relaxed lumbar spine muscles could provide better surgical conditions. Recently, a study investigating surgical conditions of lumbar surgery presented that deep NMB provided better surgical conditions with lower lumbar retractor pressure and higher overall satisfaction score evaluated by surgeons than no NMB^13^. We observed outcomes that were consistent with those reported previously; compared with group A, in the group T, which manifested deeper NMB, the overall surgical satisfaction score evaluated by the surgeon was superior, and the lumbar muscle tone and lumbar retractor pressure were lower, which resulted in better surgical conditions in group T. Hence, the acceleromyography at the trapezius could more accurately reflect the actual NMB values in the lumbar spine muscles than that of the adductor pollicis muscle.

Acceleromyography at the trapezius can establish an optimal time of tracheal intubation. Several studies have attempted to determine the appropriate measurement sites for indicating the optimal intubating time and conditions^24,25^, but measurement sites, such as the corrugator supercilii, orbicularis oculi, masseter, and mylohyoid muscles, were not commonly used because they were extremely small and the measured values could be inaccurate. Recently, the trapezius was suggested as an alternative site to the adductor pollicis muscle for guiding proper intubating conditions^11^, and consistent results for optimal intubation timing and conditions were observed in our study. Acceleromyography at the trapezius detected the optimal timing for tracheal intubation, and the outcomes for intubating conditions were comparable to those of group A. These results suggested that acceleromyography at the trapezius can indicate the optimal timing for tracheal intubation more rapidly, while providing similar intubating conditions.

Higher pressure on retractor can cause the edema and injury of lumbar spine muscles, and result in the more severe postoperative pain^13,26,27^. In our previous study, deep NMB showed a statistically significant decrease in intraoperative retraction pressure and led to lower postoperative back pain compared with no NMB^13^. However, in the present study, there was no difference in postoperative pain and analgesic consumption between the two groups, suggesting that the difference between deep and moderate NMB may not affect the degree of postoperative pain and amount of analgesic consumption in lumbar surgery. Further well-designed studies are needed to investigate the effects of deep vs. moderate NMB on postoperative pain after lumbar surgery.

Our protocol contains some limitations. First, the NMB management and monitoring in this study were performed according to the Good Clinical Research Practice guidelines^12^. Nevertheless, a tetanic stimulation for signal stabilization could not be applied for shortening the stabilization period, because tetanic stimulation with high frequency on the trapezius could be devastating by generating contraction of the innervated muscles through accidental stimulation of the nerves such as phrenic nerve near the trapezius^10^. To overcome this limitation, we are planning to perform another study at the trapezius muscle using electromyographic monitoring that does not require signal stabilization using tetanic stimulation. In the same vein, since deep NMB is assessed by post-tetanic count (PTC) with TOF stimulation, it is recommended to monitor at the adductor pollicis and to usually maintain TOF count 0 and PTC 1–2. Nevertheless, given that moderate NMB at the trapezius was sufficient to provide proper surgical conditions during spine surgery in our study, PTC stimulation for providing deep NMB at the trapezius muscle may not be necessary in the usual clinical setting. Second, the signal stabilization processes including calibration and re-calibration were performed before the administration of neuromuscular blocking agent in the supine position, but were not re-performed after the patients were turned prone for this surgery. To make the protocol possible, re-stabilization process required the full recovery of NMB and re-administration of neuromuscular blocking agent. For instance, sugammadex should be administered after turning the patients prone to reverse rocuronium-induced NMB, and then cisatracurium should be administered to maintain moderate NMB again after re-stabilization process, however, these protocols are too complex to apply to the clinical research as well as the actual clinical setting. Third, the benefit of monitoring at the trapezius during lumbar surgery with respect to surgical conditions, in essence, was originated from the more dose of rocuronium and consequent deeper depth of NMB. Therefore, similar or more surgical satisfaction will be anticipated when applying a deep block when using the adductor muscle as monitoring site. Last, surgical satisfaction assessed at only one time point after the conclusion of the procedure rather than more time points differences might cause a recall bias.

In conclusion, acceleromyographic monitoring at the trapezius muscle required a higher dose of rocuronium to maintain moderate NMB, thereby the consequent deeper degree of NMB from more rocuronium provided better surgical conditions compared to monitoring at the adductor pollicis muscle during lumbar spine surgery under general anaesthesia. Trapezius as proximal muscle may better reflect the surgical conditions of spine muscle which is manipulated during lumbar surgery.

## Supplementary Information

Supplementary Information.

## Data Availability

All data are available upon request, please contact Seok Kyeong Oh (email address: nanprayboy@korea.ac.kr).
