# Supplementary material for: Comparison between the trapezius and adductor pollicis muscles as an acceleromyography monitoring site for moderate neuromuscular blockade during lumbar surgery
Source: Sci Rep. 2021 Jul 15;11:14568. doi: 10.1038/s41598-021-94062-2 (PMC8282790; doi:10.1038/s41598-021-94062-2)

**Supplementary Figure S1.** Examples of neuromuscular monitoring at each measurement site. A: A case of the trapezius muscle group. B: A case of the adductor pollicis muscle group. Purple line: Body temperature. Calibration and stabilization were confirmed, and intubation was performed after complete block (blue arrow). After recovery to moderate neuromuscular blockade from deep neuromuscular blockade of train-of-four (TOF) count 0 (purple arrow), TOF count 1–3 was maintained based on acceleromyography at the trapezius muscle or the adductor pollicis muscle according to the allocated group (green arrow). After sugammadex injection, NMB recovered to a TOF ratio > 0.9 (red arrow).


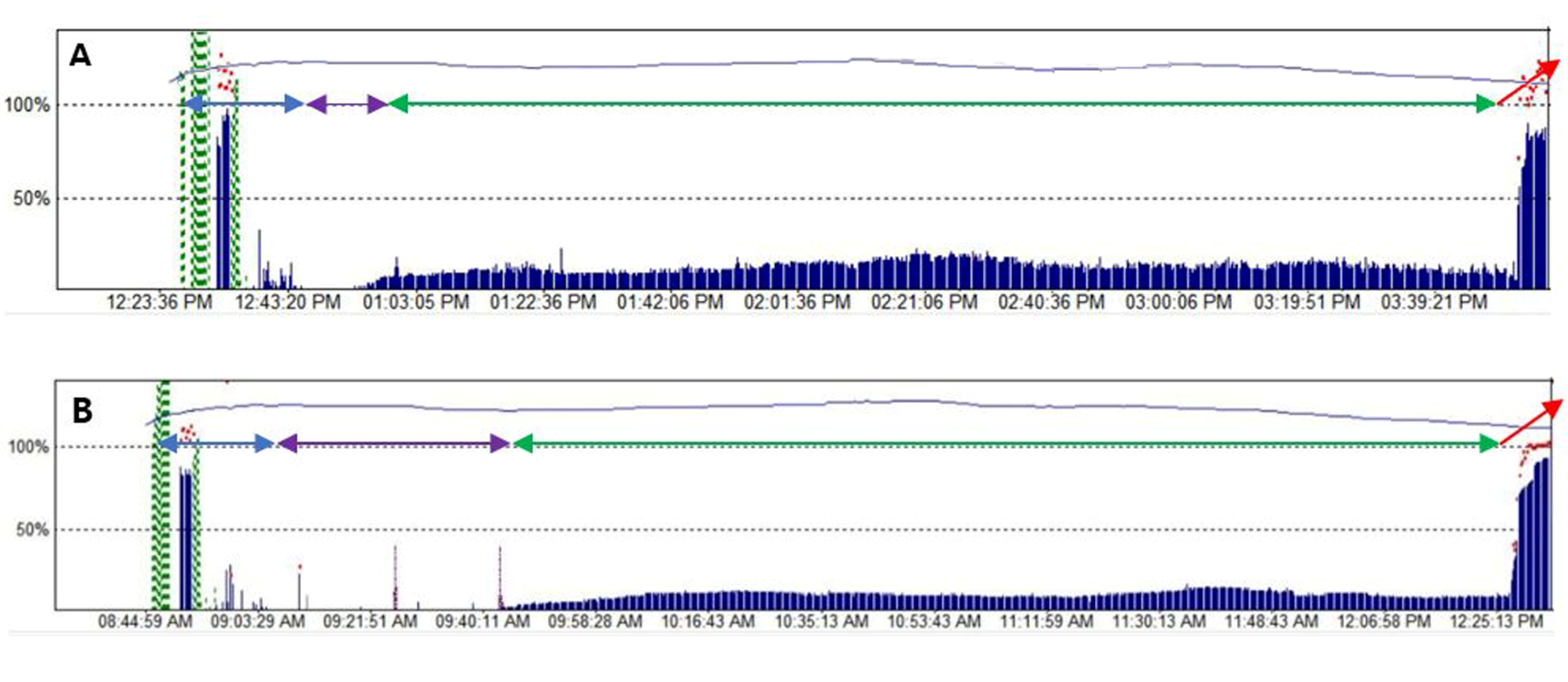

Supplement: Supplementary file 1 — Supplementary Information. [file 41598_2021_94062_MOESM1_ESM.docx]
